# Childhood maltreatment, shame, and self-esteem: an exploratory analysis of influencing factors on criminal behavior in juvenile female offenders

**DOI:** 10.1186/s40359-024-01758-x

**Published:** 2024-05-08

**Authors:** Xiaomei Chen, Bo Dai, Shuang Li, Lili Liu

**Affiliations:** 1 LuDong University, Yantai, China; 2 Guilin Municipal Government Authorities, Guilin, China; 3 Jilin Normal University, Siping, China; 4 Northwest Normal University, Lanzhou, China

**Keywords:** Childhood maltreatment, Shame, Self-esteem, Criminal behavior, Female juvenile offenders

## Abstract

**Objective:**

This study aimed to investigate the relationships between childhood maltreatment, shame, and self-esteem among juvenile female offenders and to explore the potential influencing factors on their criminal behavior.

**Methods:**

Using a stratified cluster sampling method, 1,227 juvenile female offenders from 11 provinces in China were surveyed using the Childhood Trauma Questionnaire (CTQ), Self-Esteem Scale (SES), and a self-developed Shame Questionnaire for Juvenile Offenders. Data were analyzed using descriptive statistics, correlation analysis, chi-square tests, t-tests, and structural equation modeling with mediation analysis.

**Results:**

(1) Childhood maltreatment have a significant potential influencing factors on criminal behavior; (2) Childhood maltreatment was positively correlated with self-esteem(*β* = 0.351, *p* < 0.001); (3) shame (*β* = 0.042, *p* < 0.001) mediate the relationship between Childhood maltreatment and self-esteem (childhood maltreatment → shame → self-esteem (95% Cl: 0.033, 0.052)).

**Conclusion:**

This study demonstrates that childhood maltreatment is a significant predictor of criminal behavior among juvenile female offenders. childhood maltreatment can directly influence of self-esteem, which can also affect juvenile female offenders’self-esteem indirectly through shame. The findings suggest that shame are important variables that mediate the effect of the juvenile female offenders’childhood maltreatment on their self-esteem.

## Introduction

Child maltreatment remains a pervasive global issue with far-reaching impacts that extend into every corner of society. juveniles, the future of our society, are influenced by a multitude of factors in their growth and development, one of which is their early life experiences. As emphasized by the World Health Organization, child maltreatment includes physical, emotional, and sexual abuse, neglect, and exploitation, all potentially harmful to a child’s overall health [1]. Moreover, maltreatment of children is not just about acts of commission; it also involves acts of omission, which include neglecting the emotional and physical needs of children [2]. The impact of child maltreatment on society becomes particularly evident in the context of criminal behavior. Notably, a link between experiencing abuse during childhood and delinquent behavior in adolescence has been observed [3]. According to existing literature, such traumatic experiences during formative years lead to challenges in interpersonal relationships and might result in maladaptive behaviors [4, 5]. These experiences of abuse can have lasting impacts on a child’s psychological and emotional health [6]. For instance, children who are frequently abused may exhibit serious mental problems, such as psychological trauma, depression, anxiety, suicidal tendencies, violent tendency, and aggression. Among those with a history of abuse, behaviors such as school absenteeism, risky behaviors, and even physical altercations become more prevalent [7, 8]. Simultaneously, these behaviors might be their way of coping with traumatic experiences.

In China, childhood maltreatment is a significant influencing factor in juvenile criminal behavior [9, 10]. Studying the relationship between childhood maltreatment and criminal behavior in the Chinese context is unique and necessary, mainly reflected in three aspects. First, the collectivistic cultural characteristics of China may make childhood maltreatment more easily concealed, and victims are less willing to seek help, leading to more severe psychological consequences and criminal risks. Second, China’s relatively inadequate laws and policies in preventing and dealing with childhood maltreatment may exacerbate victims’ psychological trauma and criminal risks. Finally, China’s rapid social transformation and modernization may increase the risk of childhood maltreatment while bringing more uncertainties and challenges to juveniles, increasing their psychological vulnerability and criminal risks. Research shows that among juvenile offenders, theft, group fighting, provocative disturbance, robbery, rape, and intentional injury are the most common criminal behaviors, accounting for 76.5% of all criminal behaviors [11]. Therefore, conducting research on the relationship between childhood maltreatment and criminal behavior in the Chinese context can reveal the influence of cultural characteristics, legal policies, and social transformation factors, providing important theoretical and practical basis for improving relevant policies, preventing and intervening in childhood maltreatment and its resulting criminal behavior. At the same time, it also helps to fill the research gap in this field in China and provide empirical evidence from a Chinese perspective for cross-cultural research. However, when studying juvenile delinquency, few studies focus on female juvenile offenders. They may have unique experiences and challenges that differ from male offenders [12]. The underrepresentation of female juvenile offenders in scholarly research is a significant gap that our study aims to address. Historically, the majority of studies in juvenile delinquency have centered on male populations, leading to a skewed understanding of the factors contributing to criminal behavior and the effective interventions needed for rehabilitation. This gender bias overlooks the unique socio-cultural, psychological, and environmental factors influencing female adolescents’ pathways into criminal behavior. Female juvenile offenders often face distinct challenges and vulnerabilities compared to their male counterparts. Research indicates that girls are more likely to experience certain forms of trauma, such as sexual abuse, which can have profound impacts on their psychological development and lead to different coping mechanisms, including delinquent behavior [13, 14]. Furthermore, societal norms and gender expectations can exacerbate the stigma and shame associated with female delinquency, influencing their self-esteem and identity formation [15]. Understanding the specific needs and experiences of female juvenile offenders is crucial for developing targeted interventions that address the root causes of their criminal behavior and support their rehabilitation and reintegration into society.

In recent years, there has been an increasing focus on understanding the specific relationship between childhood maltreatment and juvenile delinquency. Research findings indicate a positive correlation between these two factors, with individuals who experienced physical abuse during childhood being more likely to exhibit aggressive behavior during adolescence [16]. This highlights the importance of prevention and intervention measures targeting childhood physical abuse to mitigate its impact on later criminal activities. Related studies have explored the relationship between early traumatic experiences and criminal behavior, primarily focusing on male prisoners. However, their research findings suggest that the impact of early trauma may also have significant implications for criminal behavior among female juveniles [17]. Some researchers investigated the link between childhood emotional abuse and potential aggressive behavior in early adulthood. Their study emphasizes the importance of mentalization abilities as a mediating factor in this relationship. Mentalization refers to the ability to understand the mental states underlying human behavior, which may be crucial for female juveniles in processing the impact of emotional abuse and avoiding the development of criminal behavior [18]. Furthermore, research has highlighted the role of gender in the impact of abuse, as female participants exhibited BDSM-type sexual addiction, self-attacking behaviors, and alcohol abuse [19]. This case study emphasizes the need for gender-specific interventions and support systems for female juveniles who have experienced abuse and violence. The “life course perspective” model suggests that childhood abuse indirectly increases the propensity for criminal behavior later in life by exacerbating juvenile delinquency. When juvenile offenders are labeled by society, this initial deviance may be reenacted in later life, leading to the recurrence of criminal behavior [20].

The process of being socially labeled intensifies feelings of social exclusion, thereby reducing opportunities for behavioral correction or reintegration into society [21]. This negative cycle makes it more likely for abused juveniles to continue engaging in criminal activities in adulthood. While the life course perspective model emphasizes the indirect pathway from childhood abuse to criminal behavior in adulthood through juvenile delinquency, we must recognize that various factors can mediate the direct link between these two variables. Individual coping mechanisms, the presence of social support systems, and access to effective interventions can all play a role in mitigating the long-term negative effects of abuse [22]. This implies that not all individuals who experience childhood maltreatment will follow a criminal trajectory, as positive factors at the societal, familial, and individual levels can intervene and alter the course of criminality. The relationship between childhood maltreatment and criminal behavior among female juveniles is a complex process involving the interaction of multiple factors, encompassing both direct influences and indirect effects through juvenile delinquency. The studies reviewed in this literature review highlight the importance of considering the specific forms of maltreatment, such as physical and emotional abuse, as well as the role of gender in shaping the psychological and behavioral outcomes of maltreatment. Consistent with the proposal, we formed our hypothesis 1 as follows: Childhood maltreatment is an important influencing factor in the criminal behavior of juvenile female offenders.

Childhood maltreatment, including various forms of abuse and neglect, is considered a significant risk factor for a range of adverse outcomes, including low self-esteem. Self-esteem is defined as an individual’s overall subjective evaluation of their own worth and plays a crucial role in psychological well-being and social functioning. Shen (2009) investigated the combined impact of interparental violence and child physical abuse on juvenile self-esteem, and the results showed that both forms of abuse experienced during childhood had long-term detrimental effects on self-esteem in adulthood [23]. This finding highlights the compound impact of various forms of abuse on an individual’s self-perception and worth, emphasizing the need for comprehensive interventions targeting multiple forms of maltreatment. Additionally, The study sampled emerging adults from low socioeconomic backgrounds and examined the relationship between childhood maltreatment and various adverse psychological outcomes, including reduced self-esteem [24]. Their research adds to the literature linking childhood maltreatment to negative psychological outcomes, highlighting the importance of considering socioeconomic factors when examining the impact of abuse on self-esteem. Furthermore, study found a negative correlation between childhood maltreatment and self-esteem [25]. Researchers further explored the correlation between self-esteem and child abuse. The study found that psychological abuse and neglect were negatively correlated with self-esteem, which in turn was associated with various forms of internalizing and externalizing behavior problems [26–28]. In addition, study explored the protective role of self-related resources, such as self-esteem and self-compassion, in the relationship between childhood maltreatment and subjective well-being in early adulthood [29]. Collectively, the research findings emphasize the significant impact of childhood maltreatment on self-esteem. Based on the above discussion, by combining the aforementioned hypotheses, this study proposes the following hypothesis 2: Childhood maltreatment has a significant impact on self-esteem in juvenile female offenders.

In recent years, research on childhood shame has become increasingly rich, with numerous studies demonstrating that experiences of shame have profound effects on individual psychological health and self-perception, and are also closely related to childhood maltreatment. A study investigated the direct link between childhood maltreatment and the development of shame, demonstrating that these experiences largely contribute to subsequent shame [30], And further explored the relationship between childhood maltreatment and shame by examining how maladaptive schemas mediate this link [31]. Their research findings suggest that maladaptive schemas formed due to abuse heighten sensitivity to shame and guilt, which in turn affects emotion regulation and self-esteem. Some researchers explored the psychological pathways from childhood maltreatment to depression and crime, highlighting the process of juvenile shame transforming into guilt and self-blame [32, 33]. There are also some studies explored the broader societal impact of shame, examining its relationship with racism, social anxiety, and bullying victimization [34, 35]. These studies indicate that shame not only stems from direct abuse but can also be exacerbated by social threats to an individual’s relationships and status, further impacting self-esteem and psychological well-being. This research emphasizes the importance of considering the social context in which shame arises and its far-reaching effects on individual well-being. Several studies focused on specific populations, such as individuals with psychosis, investigating the impact of socially induced shame, self-blame, and low self-esteem [36, 37]. These studies provide deeper insights into how internalized shame and self-esteem mediate the relationship between stigma, emotional distress, and recovery in individuals with psychosis, highlighting the central role of shame in the experience of mental health challenges. The reviewed research suggests that shame plays a crucial role in the relationship between childhood maltreatment and various psychological outcomes, including self-esteem. The internalization of shame often stems from maladaptive schemas and social pressures, significantly impacting an individual’s self-esteem, emotional well-being, and behavioral patterns. Recognizing the central role of shame in these dynamics is essential for developing targeted interventions to mitigate the long-term effects of childhood maltreatment and promote resilience and recovery. Based on the above analysis, we propose hypothesis 3: Shame mediates the relationship between childhood maltreatment and self-esteem in juvenile female offenders.

In summary, this study aims to uncover childhood maltreatment as an important influencing factor in juvenile female offenders’ criminal behavior, as well as its relationship with self-esteem and shame, and to examine the mediating effect of shame in the relationship between childhood maltreatment and self-esteem, thereby analyzing the sociopsychological mechanisms of juvenile female offenders’ criminal behavior. This study plans to establish a mediation model to deeply explore the influence of sociopsychological mechanisms such as childhood maltreatment, self-esteem, and shame on the criminal behavior of juvenile female offenders. The research results will help provide targeted recommendations for the prevention and intervention of childhood maltreatment and reduce the long-term negative impact of abuse. Additionally, the research results will provide a basis for the psychological treatment and rehabilitation of female offenders, aiding in the design of intervention programs focusing on self-esteem and shame. Furthermore, this study will also provide references for reducing the risk of recidivism among female offenders and formulating effective rehabilitation and re-socialization strategies.

## Method

### Participants and procedure

China contains 681 prisons, due to the large number of prisons this study randomly selected 11 provinces prisons, which contain 3 prisons in west of China,4 prison in east of China prison and 4 central prisons of China. From June to July 2023, Paper questionnaires were distributed to juvenile female offenders in these 11 provinces, yielding a total of 1,321 responses. All of the questionnaires are received back, after selected all those questionnaires 1,227 valid responses were obtained, resulting in a questionnaire validity rate of 92.88%, the invalid questionnaires contains unclear answers and blurry messages, and deleted all those questionnaires.

Among the participants, the majority were non-only children (84.27%), with most having an educational level of junior high school or below (59.90%). The majority resided in rural areas (48.90%), came from families where the parents were in their first marriage (68.05%), and had moderate family economic conditions (56.07%). The most common offenses were sexual crimes and fraud (19.64% and 23.88%, respectively), with the majority of crimes being committed in groups (60.64%).

Before the study, informed consent was obtained from departmental and prison leaders as well as the juvenile female offenders themselves. The survey was conducted in a group format, led by two psychology postgraduate students in each prison area. A standardized introduction was used to ensure all participants clearly understood the purpose and process of the survey. The entire survey took approximately 15 min to ensure necessary information was collected efficiently. This study was approved by the Ethics Review Committee of Nanshan Hospital of Shandong Province (Approval Number: [2023-07-X105]). All of the procedures were performed in accordance with the Declaration of Helsinki and relevant policies in China. All participants agreed to participate voluntarily, with informed consent when they fled in the survey.

## Measure

### Demographic questionnaire

We used a self-compiled demographic questionnaire to survey: Only child status (Yes/No), Place of origin, Education Level, Type of Residence, Parental Marital Status(Intact/Remarried/Single Parent), Types of Crime(Property Crime/ Violent Crime/ Sexual Crime /Other), Family’s Economic Status in the Local Area(Better Off/Average/Below Average/Poor).

### Childhood trauma questionnaire (CTQ)

The Childhood Trauma Questionnaire (CTQ) developed by Bernstein (1998) [38] and later translated and modified into Chinese by Zhao Xingfu (2004) [39] was used. Designed to measure maltreatment experiences before the age of 16, this questionnaire serves as a screening tool to identify individuals with childhood abuse and neglect experiences. The questionnaire comprises five sub-questionnaires with five items each: emotional abuse, physical abuse, sexual abuse, emotional neglect, and physical neglect. Scoring ranges from “never"=1 to “always"=5. Out of 28 questions, 25 assess the questionnaire’s main components, and 3 identify individuals denying childhood issues. The total score of the sub-questionnaires ranged from 0 to 25, with higher scores indicating more severe abuse. The Cronbach’s alpha coefficients of the subscales ranged from 0.79 to 0.92, indicating good reliability.

### Self-esteem scale (SES)

The Self-Esteem Scale (SES), developed by the Rosenberg, is used to assess juveniles’ overall sense of self-worth and self-acceptance. It consists of 10 items, each rated on a four-point scale: 1 indicates “strongly agree,” 2 indicates “agree,” 3 indicates “disagree,” and 4 indicates “strongly disagree.” Items 3, 5, 8, 9, and 10 are reverse-scored. The total score ranges from 10 to 40, with higher scores indicating higher levels of self-esteem [40]. In this study, the Cronbach’s alpha coefficient for the scale was 0.86.

### Shame questionnaire for juvenile offenders

The Shame Questionnaire for Juvenile Offenders, a self-developed questionnaire, was used in this study. The questionnaire consists of 17 items, each rated on a 5-point scale ranging from 1 (completely disagree) to 5 (completely agree). It includes three dimensions: cognitive shame, emotional shame, and behavioral shame. Higher scores indicate higher levels of shame among juvenile female offenders. In this study, the fit indices of a confirmatory factor analysis model of the scale were RMSEA = 0.06, TLI = 0.90, and CFI = 0.91. The Cronbach’s alpha coefficients for the overall questionnaire and its three dimensions were 0.86, 0.82, 0.81, and 0.72, respectively. The split-half reliabilities were 0.71, 0.78, 0.77, and 0.81, respectively.

### Statistical analysis

This study has adopted IBM SPSS22.0 statistical software for all data analyses. After the questionnaires were collected, all the data have been processed as follows: (1) Exploratory factor analysis was performed on all scales by SPSS22.0; (2) internal consistency was tested for all scales by SPSS22.0; (3) the Harman single-factor method has been adopted for the common method deviation test; (4) descriptive statistics, such as statistical means (M), standard deviations (SD), maximum and minimum values, and the Cronbach’salpha were computed; (5) Pearson correlation analysis to explore the relationship between childhood maltreatment, shame, and self-esteem; (6) T-tests were used to analyze relationship between the types of crime committed and the types of childhood maltreatment experienced, chi-square analyses were performed; (7) a Structural Equation Modelling (SEM) approach was employed to test the theoretical model in the current study. PROCESS version 3.3 macro was used to construct the structural equations and to test the mediating effects [41]. The accepted level of significance was *p* < 0.05.

## Results

### Data processing and common method bias test

In this study, common method bias was controlled through anonymous surveys and reverse scoring of some items. To further assess this bias, Harman’s single factor analysis method was used. The analysis revealed five factors without rotation, accounting for 59.95% of the total variance. The first factor explained 33.41% of the variance, below the 40% threshold, indicating that the data were not significantly affected by common method bias [42].

### Descriptive statistics of variables

The study participants consisted of 1,227 female juveniles deprived of liberty due to various criminal offenses, as recorded in the reviewed files. Table 1 reveals that the most common type of offense was property crime, accounting for 33.98% (*n* = 417), followed by sexual crimes at 26.49% (*n* = 325), violent crimes at 7.91% (*n* = 97), and other types of crimes at 31.62% (*n* = 388). However, the majority of these juveniles were exposed to negative elements within their marginalized family and educational environments. As depicted in Table 1, the percentage of family context issues (parental marital status and domestic abuse) was higher than other contexts. This was followed by school educational context (dropout rates), and lastly socio-economic context (poor economic conditions and living in marginalized and inappropriate environments), indicating that these risk factors contribute to the criminal behavior among female juvenile offenders.( Table 1).

**Table 1:** Descriptive statistics of crime types and risk factors

	Variable	Frequency	Percentage
Types of Crime	Property Crime	417	33.98
	Sexual Crime	325	26.49
	Violent Crime	97	7.91
	Other	388	31.62
Risk Factors	Family	Present 712	58.03
		Absent 515	41.97
	School	Present 674	54.93
		Absent 553	45.07
	Economy	Present 650	52.97
		Absent 577	47.03

Note: Crimes are categorized into four types based on the nature of the object harmed by the criminal activity: property crimes, violent crimes, sexual crimes, and others. Property crimes include theft, robbery, fraud, embezzlement of funds, etc.; sexual crimes encompass rape, prostitution, organizing prostitution, etc.; violent crimes cover intentional injury, murder, etc.; others include crimes such as using cult organizations to obstruct law enforcement, drug trafficking, illegal collection of public deposits, bribery by non-governmental personnel, organizing and leading pyramid schemes, using superstition to disrupt law enforcement, causing traffic accidents, provoking trouble, and other similar offenses

### Types and degrees of childhood maltreatment in female juvenile offenders

Table 2 presents the percentage distribution of types of childhood maltreatment based on the degree experienced by the female juvenile offenders studied. Significant percentages were observed at moderate and high levels, indicating the presence of maltreatment among these juveniles. Indeed, in Table 2, it can be seen that there is a prevalence of moderate to high degrees of emotional abuse, sexual abuse, physical neglect, and emotional neglect among the female juvenile offenders.

**Table 2:** Descriptive statistics of types of childhood maltreatment

Variable	Min	Max	M	SD	Skew	Kurt
Emotional Abuse	5	25	8.39	3.97	1.720	3.148
Physical Abuse	4	25	6.56	3.21	2.681	7.514
Sexual Abuse	5	25	7.29	2.17	2.261	10.328
Emotional Neglect	5	25	11.35	5.60	0.593	-0.708
Physical Neglect	5	25	8.89	3.93	1.015	0.454
Childhood Maltreatment	28	113	49.88	13.04	1.430	2.270

### Comparison between types of crime and types of childhood maltreatment

Table 3 presents the results of the analysis of differences between types of crime and types of childhood maltreatment. Significant differences were found among the four groups in terms of emotional abuse (*p* < 0.001), physical abuse ( *p* < 0.05), emotional neglect *p* < 0.01), and physical neglect ( *p* < 0.05). However, no significant difference was observed for sexual abuse (*p* > 0.05). The effect sizes (*η2*) for these differences were small, ranging from 0.004 to 0.015. The violent crime group had the highest mean scores for emotional abuse, physical abuse, emotional neglect, and physical neglect compared to the other crime type groups.

**Table 3:** Analysis of differences between types of crime and types of childhood maltreatment

Variable	Types of Crime						
	Property Crime (*n* = 417)	Violent Crime (*n* = 97)	Sexual Crime (*n* = 325)	Other (*n* = 388)			
	M	M	M	M	F	*P*	η^2^
Emotional Abuse	7.88	9.09	8.99	8.16	6.116	0.000	0.015
Physical Abuse	6.30	6.91	7.03	6.40	3.374	0.018	0.008
Sexual Abuse	7.18	7.60	7.22	7.26	1.661	0.174	0.004
Emotional Neglect	10.73	12.19	12.23	11.11	4.907	0.002	0.012
Physical Neglect	8.55	9.53	9.33	8.72	3.659	0.012	0.009

Note: *p < 0.05;**p < 0.01; ***p < 0.001; *η*^2^ is an indicator for measuring effect size

### Chi-square analysis of self-esteem, shame, and crime types

Table 4 presents the results of the chi-square analysis of self-esteem, shame, and crime type among juvenile female offenders. The participants were categorized into four groups based on their crime types. Significant differences were found among the four groups in terms of self-esteem (t = 4.36, *p* < 0.01) and shame (t = 3.59, *p* < 0.01). The violent crime group had the highest mean scores for both self-esteem and shame compared to the other crime type groups. The property crime group had the lowest mean scores for self-esteem and shame. The results suggest that juvenile female offenders who committed violent crimes tend to have higher levels of self-esteem and shame compared to those who committed other types of crimes.

**Table 4:** Chi-square analysis of self-esteem, shame, and crime types among juvenile female offenders

Variable	Property Crime	Sexual Crime	Violent Crime	Other	*t*
	M ± SD	M ± SD	M ± SD	M ± SD	
Self-Esteem	21.21 ± 5.28	22.14 ± 4.97	22.56 ± 4.46	21.39 ± 5.18	4.36^**^
Shame	19.19 ± 6.87	20.03 ± 6.71	20.94 ± 6.69	19.16 ± 8.42	3.59^**^

Note: *p < 0.05;**p < 0.01; ***p < 0.001

### Correlation analysis of childhood maltreatment, self-esteem, and shame

Table 5 presents the results of the correlation analysis of childhood maltreatment, self-esteem, and shame among juvenile female offenders. The analysis revealed significant positive correlations among various types of childhood maltreatment. However, sexual abuse was negatively correlated with emotional neglect (*r* = -0.185, *p* < 0.001) and not significantly correlated with physical neglect (*r* = -0.053, *p* > 0.05). Childhood maltreatment was positively correlated with self-esteem (*r* = 0.351, *p* < 0.001) and shame (*r* = 0.330, *p* < 0.001). Self-esteem was also positively correlated with shame (*r* = 0.414, *p* < 0.001). These findings suggest that juvenile female offenders who experienced higher levels of childhood maltreatment tend to have higher levels of self-esteem and shame. The results also indicate that different types of childhood maltreatment are interrelated, and they collectively contribute to the development of self-esteem and shame among juvenile female offenders. This result confirms Hypothesis 2.

**Table 5:** Correlation analysis of childhood maltreatment, self-esteem, and shame among juvenile female offenders

Variable	1	2	3	4	5	6	7
1 Emotional Abuse	-						
2 Physical Abuse	0.661^***^	-					
3 Sexual Abuse	0.146^***^	0.247^***^	-				
4 Emotional Neglect	0.483^***^	0.391^***^	-0.185^***^	-			
5 Physical Neglect	0.583^***^	0.484^***^	-0.053	0.776^***^	-		
6 Childhood Maltreatment	0.802^***^	0.760^***^	0.263^***^	0.760^***^	0.824^***^	-	
7 Self-Esteem	0.341^***^	0.221^***^	-0.027	0.332^***^	0.331^***^	0.351^***^	-
8 Shame	0.351^***^	0.247^***^	0.063^*^	0.224^***^	0.252^***^	0.330^***^	0.414^***^

Note: *p < 0.05;**p < 0.01; ***p < 0.001

### Mediation analysis

**Fig. 1:**
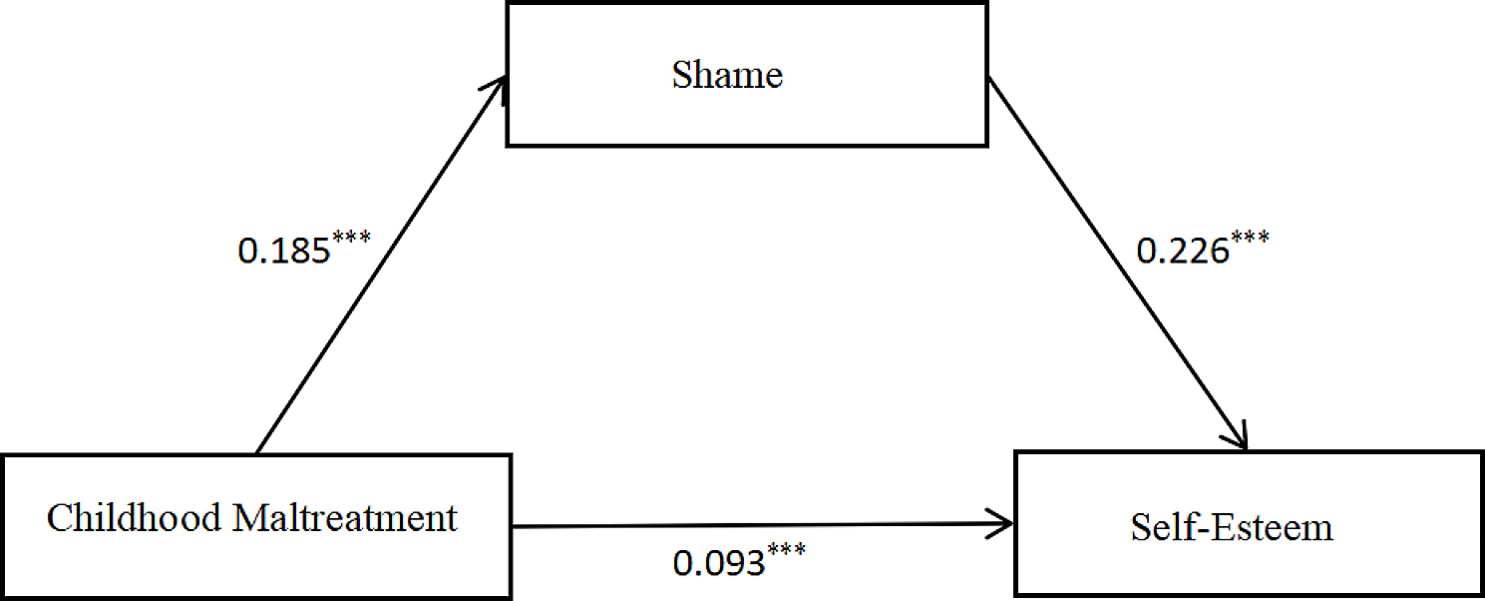
Diagram of structural equation model

The analysis of the mediation effects of exercise imagery showed that the mediation effect of shame was 0.042, and its bootstrap 95% confidence interval did not contain 0 (0.033, 0.052), which indicates that its mediation effect was significant (see Table 6; Fig. 1), This result confirms Hypothesis 3.

**Table 6:** Mediation effect analysis

Path	Effect	SE	Boot LL CI	Boot UL CI	Relative mediation effect
Mediating effect	0.042	0.005	0.033	0.052	18%
Direct effect	0.093	0.011	0.072	0.014	41%
Total effect	0.135	0.011	0.114	0.114	59%

## Discussion

Child abuse, encompassing neglect and harm to children under 18, includes emotional abuse, physical abuse, neglect, and sexual abuse. Emotional neglect occurs when a child’s emotional and relational needs are unmet due to a lack of attention. Emotional abuse involves inappropriate behaviors that disrespect a child’s relationships with others, potentially negatively impacting their psychological and emotional development. Additionally, physical neglect includes inadequate and unsafe supervision of minors, potentially placing them in danger and even predisposing some juveniles to criminal pathways [43]. This study conducted an explorative analysis of 1,227 female juveniles deprived of liberty for various offenses, aiming to explore the relationship between their experiences of childhood maltreatment and subsequent criminal behavior. The study found a close association between childhood experiences of maltreatment, particularly emotional abuse, physical abuse, and emotional neglect, and criminal behavior in juvenile females. Significant differences were observed in the levels of emotional abuse, physical abuse, and emotional neglect across different types of criminal activities, indicating that the severity of maltreatment may influence the inclination of young women to commit various types of crimes. Specifically, property crimes, violent crimes, and other types of offenses showed significant correlations with all forms of childhood maltreatment. These findings align with domestic and international research [44–46], further confirming the impact and formation process of childhood maltreatment on juvenile criminal behavior.

The results of this study indicate that different types of childhood maltreatment experiences are prevalent among juvenile female offenders. Emotional neglect had the highest mean score, suggesting that this type of maltreatment was the most common in the sample. The positive skewness of all maltreatment types indicates that most participants reported lower levels of maltreatment experiences, while a few individuals experienced more severe maltreatment. Sexual abuse had the highest skewness and kurtosis values, indicating that the distribution of sexual abuse experiences was more uneven compared to other types of maltreatment, with a few participants reporting extremely high levels of sexual abuse. These findings are consistent with previous research, demonstrating that childhood maltreatment is common among female offenders [47]. Studies have also shown that different types of maltreatment may have distinct impacts on an individual’s developmental trajectory [48]. For example, sexual abuse may be associated with more severe mental health problems and a higher risk of criminal behavior [49]. Therefore, it is crucial to consider the maltreatment experiences of female offenders and their potential differential effects when working with this population.

This study found significant differences in the types and severity of childhood maltreatment experienced by juvenile female offenders across different crime types. The violent crime group had the highest mean scores on most maltreatment types, suggesting that this group may have experienced more severe maltreatment. This finding is consistent with previous research indicating an association between childhood maltreatment experiences and violent offending [50]. Violent offenders may have internalized aggressive behavior through social learning processes, or maltreatment experiences may have led to difficulties in emotion regulation and impulse control, increasing the risk of violent behavior [51]. However, no significant differences were found in sexual abuse, which is inconsistent with some previous studies that have shown an association between sexual abuse and sexual offending [52]. This discrepancy may be due to differences in sample characteristics or measurement methods. Future research should further explore the relationship between sexual abuse and sexual offending and the potential moderating factors that may influence this relationship. Although there were significant differences between crime type groups, the effect sizes were small, suggesting that maltreatment experiences may be just one of many factors influencing criminal behavior. Other factors, such as individual characteristics, family dynamics, peer influences, and community contexts, may also play important roles in the development of female criminal behavior [53]. Therefore, prevention and intervention efforts should adopt a multifaceted approach that addresses not only maltreatment issues but also other relevant risk and protective factors.

The results of this study indicate that juvenile female offenders of different crime types differ significantly in self-esteem and shame. The violent crime group had the highest mean scores on self-esteem and shame, while the property crime group had the lowest scores. This finding is partially consistent with previous research suggesting that violent offenders may have higher self-esteem [54]. The high self-esteem of violent offenders may serve as a defensive mechanism to cope with feelings of shame and guilt or may reflect positive attitudes toward aggressive behavior [55]. However, the violent crime group also reported higher levels of shame, which is inconsistent with some previous studies that have shown a negative association between shame and aggressive behavior [56]. This discrepancy may reflect the multifaceted nature of shame, which can either promote or inhibit aggressive behavior, depending on how individuals cope with shame [57]. For some violent offenders, high shame may lead to aggressive behavior as a way to externalize their shame. Future research should further explore the complex relationship between shame and violent offending and the potential moderating factors that may influence this relationship. The property crime group had the lowest scores on self-esteem and shame, suggesting that this group may have unique difficulties in emotional regulation. Low self-esteem and low shame may reflect negative evaluations of self-worth and a lack of concern for the consequences of criminal behavior. Prevention and intervention efforts should focus on enhancing self-esteem among property offenders while fostering healthy shame and empathy.

This study explored the relationships between childhood abuse, self-esteem, and shame, and the results revealed significant positive correlations among these variables. This finding is partially consistent with previous research, which has shown a positive correlation between childhood abuse and shame [58]. Abusive experiences may lead individuals to form negative self-evaluations and internalize shame [59]. However, the positive correlation between childhood abuse and self-esteem is inconsistent with most prior studies, which have found associations between childhood abuse and low self-esteem [60]. The positive correlation in the current study may reflect a defensive form of high self-esteem, a fragile and unstable form of self-esteem that appears as narcissism on the surface but hides deeper insecurities and self-doubts [61]. This defensive high self-esteem may serve as a coping mechanism to deal with the emotional pain and shame resulting from abusive experiences. The positive correlation between self-esteem and shame further supports the concept of defensive high self-esteem. Individuals with defensive high self-esteem may be more prone to experiencing shame because their self-esteem is built on an unstable foundation, making it vulnerable to threats and challenges [55]. When faced with difficulties or failures, they may be more likely to interpret these as reflections of their own deficiencies or inadequacies, triggering feelings of shame. These findings highlight the complex impact of childhood abuse on the emotional well-being of juvenile female offenders. While abusive experiences may lead to a superficially high self-esteem, this self-esteem may be fragile and defensive, associated with greater shame. Prevention and intervention efforts should focus on fostering genuine self-esteem, one that is based on self-acceptance and a sense of self-worth, rather than reliance on external validation [62].

The results of the mediation analysis indicate that childhood abuse affects self-esteem through two pathways: The direct effect suggests that experiences of childhood abuse may lead to increased self-esteem. The mediation effect suggests that childhood abuse also indirectly influences self-esteem by increasing feelings of shame. This finding supports the theoretical perspective that shame plays a crucial role in the impact of childhood abuse on self-esteem [59]. Childhood abuse may first evoke intense feelings of shame, which in turn may lead to changes in self-esteem. Shame may prompt individuals to adopt defensive strategies, such as displaying an inflated sense of self-esteem, to cope with painful emotions and self-doubt [55]. The study results provide valuable information for formulating prevention and intervention measures targeting female juvenile offenders. Particularly in family and educational environments, more attention and resources are needed to mitigate these environmental factors’ negative impacts on young women and to provide necessary support and treatment for those who have already suffered abuse.

### Practical implications

The findings of this study have important practical implications for the prevention and treatment of criminal behavior in juvenile female offenders with a history of childhood maltreatment. The results emphasize the necessity of early identification and intervention for abused children, the importance of incorporating shame-reduction and self-esteem building strategies into treatment programs, and the need for a comprehensive treatment approach. Furthermore, the study highlights the need for further research to develop and evaluate targeted interventions for this population, which may help reduce recidivism rates and improve long-term outcomes.

### Limitations and future directions

The study’s limitations include reliance on self-report measures, potential recall or reporting bias, and a cross-sectional design that precludes causal inferences. Future research should incorporate diverse data sources, employ longitudinal designs, and investigate additional correlates of criminal behavior to gain a more comprehensive understanding of female offending. The role of defensive high self-esteem in the relationship between childhood maltreatment and maladaptive outcomes, as well as potential moderating factors, warrant further exploration. Despite these limitations, the findings emphasize the importance of addressing childhood maltreatment in prevention and intervention efforts for female offenders. Trauma-informed care approaches may promote rehabilitation and reduce recidivism risk. Further research is needed to develop effective strategies fostering the healthy development of childhood maltreatment survivors.

## Conclusions

This study investigated the relationships between childhood maltreatment, shame, and self-esteem among juvenile female offenders and explored the potential influencing factors on their criminal behavior. Notably, the study found that childhood maltreatment was positively associated with both shame and self-esteem, suggesting that abusive experiences may lead to a defensive form of high self-esteem that masks underlying insecurities and self-doubt. Mediation analysis further indicated that childhood maltreatment affects self-esteem through direct and indirect pathways. The direct effect suggests that childhood abuse may lead to increased self-esteem, possibly reflecting a defensive coping mechanism. The indirect effect, mediated by shame, suggests that childhood maltreatment may first evoke intense feelings of shame, which in turn influence self-esteem. Shame may prompt individuals to adopt defensive strategies, such as displaying an inflated sense of self-esteem, to cope with painful emotions and self-doubt.

## Data Availability

No datasets were generated or analysed during the current study.
